# Analysis of Mitochondrial Calcium Retention Capacity in Cultured Cells: Permeabilized Cells Versus Isolated Mitochondria

**DOI:** 10.3389/fphys.2021.773839

**Published:** 2021-12-07

**Authors:** Sehwan Jang, Xavier R. Chapa-Dubocq, Silvia Fossati, Sabzali Javadov

**Affiliations:** ^1^Department of Physiology, University of Puerto Rico School of Medicine, San Juan, PR, United States; ^2^Alzheimer’s Center at Temple, Lewis Katz School of Medicine, Temple University, Philadelphia, PA, United States

**Keywords:** permeabilized cells, mitochondria, calcium retention capacity, permeability transition pore, mitochondrial swelling

## Abstract

In response to various pathological stimuli, such as oxidative and energy stress accompanied by high Ca^2+^, mitochondria undergo permeability transition (PT) leading to the opening of the non-selective PT pores (PTP) in the inner mitochondrial membrane. Opening of the pores at high conductance allows the passage of ions and solutes <1.5 kD across the membrane, that increases colloid osmotic pressure in the matrix leading to excessive mitochondrial swelling. Calcium retention capacity (CRC) reflects maximum Ca^2+^ overload of mitochondria that occurs just before PTP opening. Quantification of CRC is important for elucidating the effects of different pathological stimuli and the efficacy of pharmacological agents on the mitochondria. Here, we performed a comparative analysis of CRC in mitochondria isolated from H9c2 cardioblasts, and in permeabilized H9c2 cells *in situ* to highlight the strengths and weaknesses of the CRC technique in isolated cell mitochondria vs. permeabilized cells. The cells were permeabilized by digitonin or saponin, and the Ca^2+^-sensitive fluorescence probe Calcium Green-5N was used in both preparations. Results demonstrated the interference of dye-associated fluorescence signals with saponin and the adverse effects of digitonin on mitochondria at high concentrations. Analysis of the CRC in permeabilized cells revealed a higher CRC in the saponin-permeabilized cells in comparison with the digitonin-permeabilized cells. In addition, the mitochondrial CRC in saponin-permeabilized cells was higher than in isolated mitochondria. Altogether, these data demonstrate that the quantification of the mitochondrial CRC in cultured cells permeabilized by saponin has more advantages compared to the isolated mitochondria.

## Introduction

Mitochondria are the cell powerplants that provide over 90% of ATP required for cell metabolism. Also, mitochondria play a pivotal role in the maintenance of ion homeostasis, cell growth, redox signaling, and cell death. The metabolism and function of mitochondria are regulated by changes in the matrix volume associated with ion fluxes, particularly Ca^2+^, across the inner mitochondrial membrane. At low concentrations, Ca^2+^ induces negligible matrix swelling (Ichas and Mazat, 1998) and stimulates mitochondrial bioenergetics through fatty acid oxidation, tricarboxylic acid cycle, and oxidative phosphorylation (Halestrap, 1994; Tarasov et al., 2012). However, high Ca^2+^ causes excessive mitochondrial swelling leading to mitochondrial dysfunction and cell death. The main mechanism of mitochondrial swelling involves the opening of non-selective channels known as permeability transition pores (PTPs) in low-conductance (physiological) and high-conductance (pathological) modes in the inner mitochondrial membrane (Ichas and Mazat, 1998; Kwong and Molkentin, 2015). Mitochondrial swelling is driven by a high colloid osmotic pressure in the matrix of mitochondria exerted by high Ca^2+^ and non-diffusible matrix proteins. Quantification of the extent of mitochondrial swelling is important for the analysis of mitochondrial damage in response to various pathological stimuli.

The Ca^2+^ retention capacity (CRC) is broadly used to quantify the extent of PTP opening since CRC indicates the maximum Ca^2+^ uptake that mitochondria reach before PTP opening. Therefore, the amount of external Ca^2+^ that induces Ca^2+^ release through the PTP reflects the CRC of mitochondria that corresponds to the maximum mitochondrial swelling. The CRC can be quantified in isolated mitochondria and intact cells (without isolation of mitochondria). Although the fundamental knowledge on the structural organization, metabolism, and function of mitochondria, as well as their response to a wide range of diseases, was acquired using isolated cell or tissue mitochondria, the use of isolated mitochondria has several disadvantages (Kuznetsov et al., 2008; Dedkova and Blatter, 2012; Salabei et al., 2014). First, the isolation of mitochondria requires a relatively large quantity of cells or tissues since some parts are lost in the isolation process (centrifugation and washing). Second, isolated mitochondria do not represent all populations of mitochondria since mitochondria localized in certain subcellular compartments (e.g., intrafibrillar mitochondria) are not isolated by homogenization. Third, mitochondria, especially dysfunctional (fragile) mitochondria from pathological cells/tissues are partially damaged or lost during the isolation process. Fourth, isolation from the essential intracellular environment has severe effects on the morphology, metabolism, and function of mitochondria and changes their sensitivity to exogenous factors.

In this study, we evaluated the CRC in intact cells *in situ* permeabilized by two different biological detergents and in mitochondria isolated from the cultured cells to clarify the advantages and disadvantages of each technique.

## Materials and Methods

### Animals

Adult Sprague Dawley male rats (275–325 g) were purchased from Taconic (Hillside, NJ, United States). All experiments were performed according to protocols approved by the UPR Medical Sciences Campus Institutional Animal Care and Use Committee and conformed to the National Research Council Guide for the Care and Use of Laboratory Animals published by the US National Institutes of Health (2011, eighth edition).

### Cells

H9c2 embryonic rat cardioblastic cells were cultured according to the manufacturer’s recommendations (American Type Culture Collection, Manassas, VA). Briefly, the cells were cultured in DMEM based modified media containing 4 mML-glutamine, 4.5 g/L glucose, 1 mM sodium pyruvate, and 1.5 g/L sodium bicarbonate supplemented with 10% fetal bovine serum and 1% antibiotic solution (HyClone) and maintained in 95% air and 5% CO2 at 37°C. Cells maintained within 80–90% confluence from passages 3–10 were used for experiments.

### Isolation of Mitochondria From Rat Hearts

The isolation of mitochondria was adopted and modified from previous studies (Chapa-Dubocq et al., 2020). Briefly, heart ventricles were cut and homogenized using a Polytron homogenizer in ice-cold sucrose buffer containing (in mM): 300 sucrose, 20 Tris–HCl, and 2 EGTA, pH 7.2, and supplemented with 0.05% BSA. The heart homogenate was centrifuged at 2000 × *g* for 3 min to remove cell debris. The supernatant was centrifuged at 10,000 × *g* for 6 min to precipitate mitochondria and then washed again under the same conditions in sucrose buffer (BSA-free). The final pellet containing mitochondria was resuspended in 300 μl of sucrose buffer.

### Isolation of Mitochondria From Cells

To isolate mitochondria from cultured cells, H9c2 cells were trypsinized and pelleted at 200 × *g* for 7 min (Jang and Javadov, 2018). Pellet was resuspended in ice-cold sucrose buffer containing (in mM): 300 sucrose, 10 Tris–HCl, and 2 EGTA; pH 7.4. Cells were centrifuged at 2,500 × *g* for 5 min at 4°C, the pellet was resuspended in the sucrose buffer. To disrupt the plasma membrane and expose mitochondria, cells were plunged using a 27G needle until all cells were successfully lysed. The cell lysate was then centrifuged at 400 × *g* for 5 min and the supernatant was collected. The mitochondria were concentrated by centrifugation at 10,000 × *g* for 5 min and finally dissolved with sucrose buffer.

### Permeabilization of Cells

The basic principles of the cell permeabilization technique for analysis of mitochondrial function *in situ* have been described in detail elsewhere (Kuznetsov et al., 2008; Dedkova and Blatter, 2012; Salabei et al., 2014). Cells were freshly harvested using trypsin–EDTA then permeabilized in sucrose buffer (300 mM sucrose, 10 mM Tris–HCl, 2 mM EGTA, pH 7.4) containing saponin or digitonin for 10 min on ice. After the permeabilization, cells were washed with equilibration buffer (100 mM sucrose, 10 mM Tris–HCl, 10 μM EGTA, pH 7.4), then resuspended in incubation buffer (200 mM sucrose, 10 mM Tris-MOPS, 5 mM α-ketoglutarate, 2 mM malate, 1 mM Pi, 10 μM EGTA-Tris, pH 7.4) containing 100 nM Calcium Green-5N.

### Fluorescence Imaging

To evaluate the effects of permeabilization on mitochondrial function, live H9c2 cells grown to 70–80% confluence were treated with saponin or digitonin for 10 min. Then, the cells were further incubated for 30 min with 300 nM DAPI for visualization of the nucleus and 30 nM Mitotracker Red, a membrane potential-dependent dye. As a positive control, 0.1% Triton X-100 was applied to induce irreversible permeabilization of the plasma membrane leading to structural collapse of the cells. Cell images were captured by an Olympus IX73 microscope with LUCPLFLN10X objective using Cellsense Dimension (Olympus) software.

### CRC Assay

The CRC was quantified by the Ca^2+^-sensitive fluorescence dyes Oregon Green 488 BAPTA-1, Calcium Green-5N, or Fluo-5N that measure extramitochondrial Ca^2+^ fluorescence in the assay buffer. Briefly, freshly isolated mitochondria (0.5 mg/ml) or permeabilized fresh cells were incubated at 37°C in 0.1 ml of incubation buffer (200 mM sucrose, 10 mM Tris-MOPS, 5 mM α-ketoglutarate, 2 mM malate, 1 mM Pi, 10 μM EGTA-Tris, pH 7.4) containing one of the fluorescence dyes. Calcium was added to increase matrix Ca^2+^ load and the fluorescence intensity was recorded by CLARIOStar microplate reader (BMG Labtech).

### Mitochondrial PTP Opening

The swelling of mitochondria as an indicator of PTP opening in the presence or absence of Ca^2+^ was determined freshly isolated mitochondria (50 μg) by monitoring the decrease in light scattering at 525 nm as previously described, with minor modifications (Rodríguez-Graciani et al., 2020). The swelling buffer contained (in mM): 125 KCl, 20 MOPS, 10 Tris–HCl, 0.001 EGTA, and 2 KH_2_PO_4_, pH 7.1. The swelling curves were normalized to control and presented as an absorbance ratio.

### Statistical Analysis

Data values are presented as mean ± SE. Student’s *t*-test was used to compare differences between two groups. *p* < 0.05 was considered as statistically significant. The number of biological samples but not technical replicates were used as a sample size.

## Results

### Analysis of Mitochondrial CRC by Different Ca^2+^-Sensitive Fluorescent Dyes

**Figure 1:**
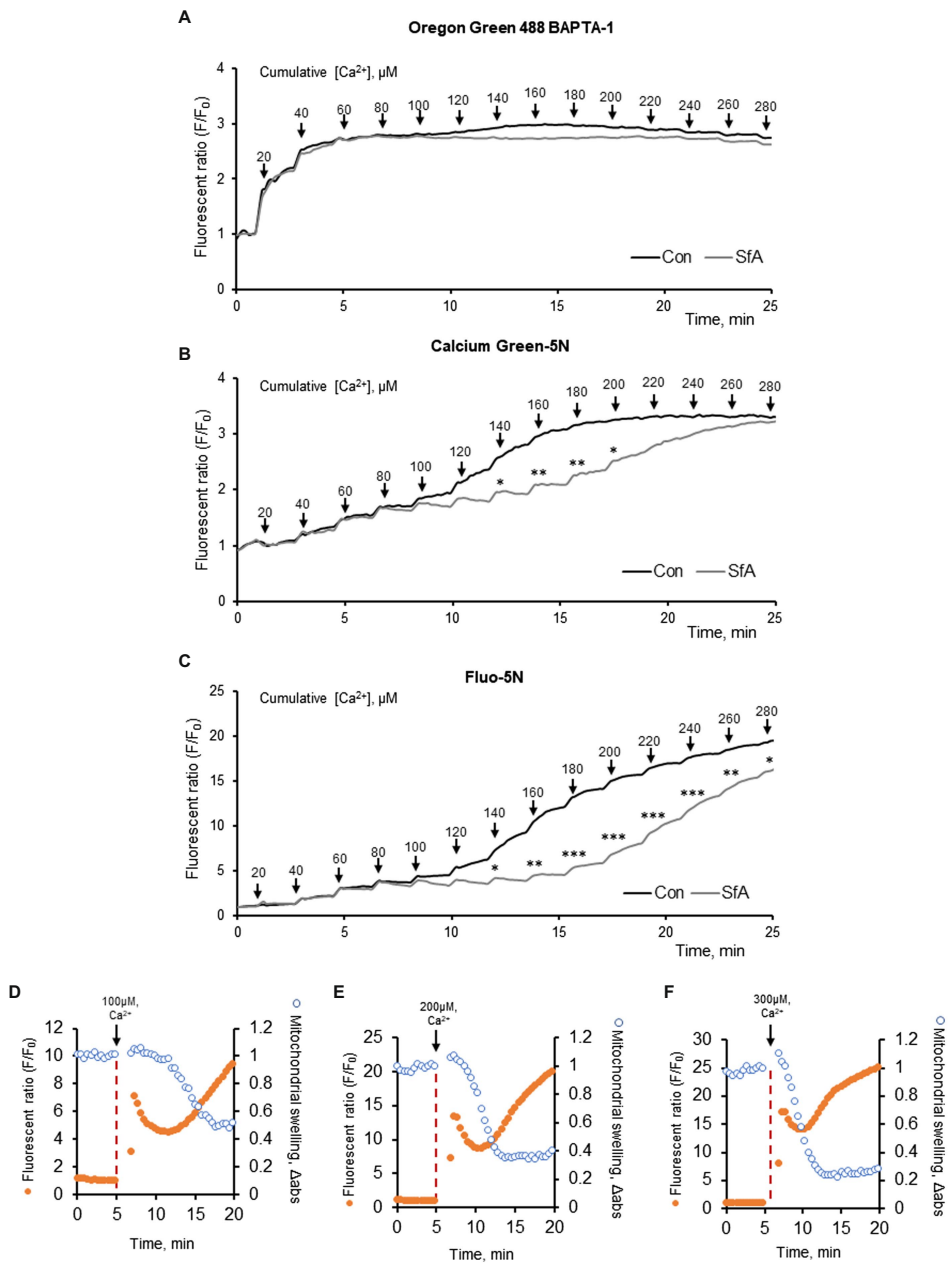
CRC analysis in mitochondria isolated from adult Sprague Dawley rat hearts. The CRC was measured using the Ca^2+^ sensitive fluorescent dyes, 100 nM Oregon Green 488 BAPTA-1 **(A)**, 100 nM Calcium Green-5N **(B)**, and 500 nM Fluo-5N **(C)**. Mitochondria (50 μg) were exposed to repetitive application of 20 μM (2 nmol) of Ca^2+^ every 2 min (*arrows*) to increase matrix Ca^2+^ load. The PTP dependence of the CRC was determined by adding 0.5 μM sanglifehrin A (SfA, a cyclophilin D inhibitor) to the incubation media. *p < 0.05, **p < 0.01, ***p < 0.001 vs. control (Con). *n* = 3 per group. **(D–F)** CRC and mitochondrial swelling analysis in mitochondria isolated from adult Sprague Dawley rat hearts. The CRC was measured using the Ca^2+^ sensitive fluorescent dyes, 500 nM Fluo-5N. Mitochondria (50 μg) were exposed to 100 **(D)**, 200 **(E)**, and 300 **(C)** μM (10, 20, 30 nmol) of Ca^2+^ at 5 min (arrows) to increase matrix Ca^2+^ load. *n* = 3 per group.

In the first set of experiments, we assessed the sensitivity of three different fluorescent dyes to Ca^2+^ in isolated heart mitochondria. The CRC was measured in 50 μg of mitochondria by using Oregon Green 488 BAPTA-1, Calcium Green-5N or Fluo-5N that possess a dissociation constant (K_d_ for Ca^2+^) of 0.17 μM, 15 μM, or 90 μM, respectively (Figure 1). Results exhibited a different sensitivity of the dyes to Ca^2+^. The Oregon Green 488 BAPTA-1 dye was the most sensitive and CRC reached the maximum at ~60 μM Ca^2+^ (Figure 1A) whereas Calcium Green-5N demonstrated the maximum CRC at 200 μM. (Figure 1B). The CRC of mitochondria was less sensitive to Fluo-5N and could not reach the maximum at 280 μM (Figure 1A). Moreover, inhibition of the mitochondrial swelling by sanglifehrin A, a PTP blocker that inhibits cyclophilin D activity, affected differently the fluorescence intensity of the dyes; inhibition of the CRC was more obvious in the presence of Calcium Green-5N or Fluo-5N (Figures 1B,C) suggesting that the inhibitory effect of sanglifehrin A on mitochondrial swelling depends on the sensitivity of the dyes to Ca^2+^ as well as on the concentration of Ca^2+^. It should be noted that decreased CRC was associated with increased PTP opening in the isolated cardiac mitochondria (Figures 1D–F). Ca^2+^ induces inner membrane expansion and outer membrane rupture (Strubbe-Rivera et al., 2021), leading to the swelling of mitochondria. The increase in matrix volume is accompanied by a decrease in the intensity of light scattered (Tedeschi and Harris, 1955). Thus, these data demonstrate that due to differences in K_d_ for Ca^2+^, the different Ca^2+^-sensitive fluorescent dyes can be applied for measurement of the CRC at low (Oregon Green 488 BAPTA-1: *K_d_* = 170 nM), medium (Calcium Green-5N: *K_d_* = 14 μM), or high (Fluo-5N: *K_d_* = 90 μM) concentrations of Ca^2+^. Moreover, we experimentally demonstrated the relationship between mitochondrial swelling and Ca^2+^ efflux in response to Ca^2+^-induced PTP opening.

### The Effects of Permeabilization on Mitochondria

**Figure 2:**
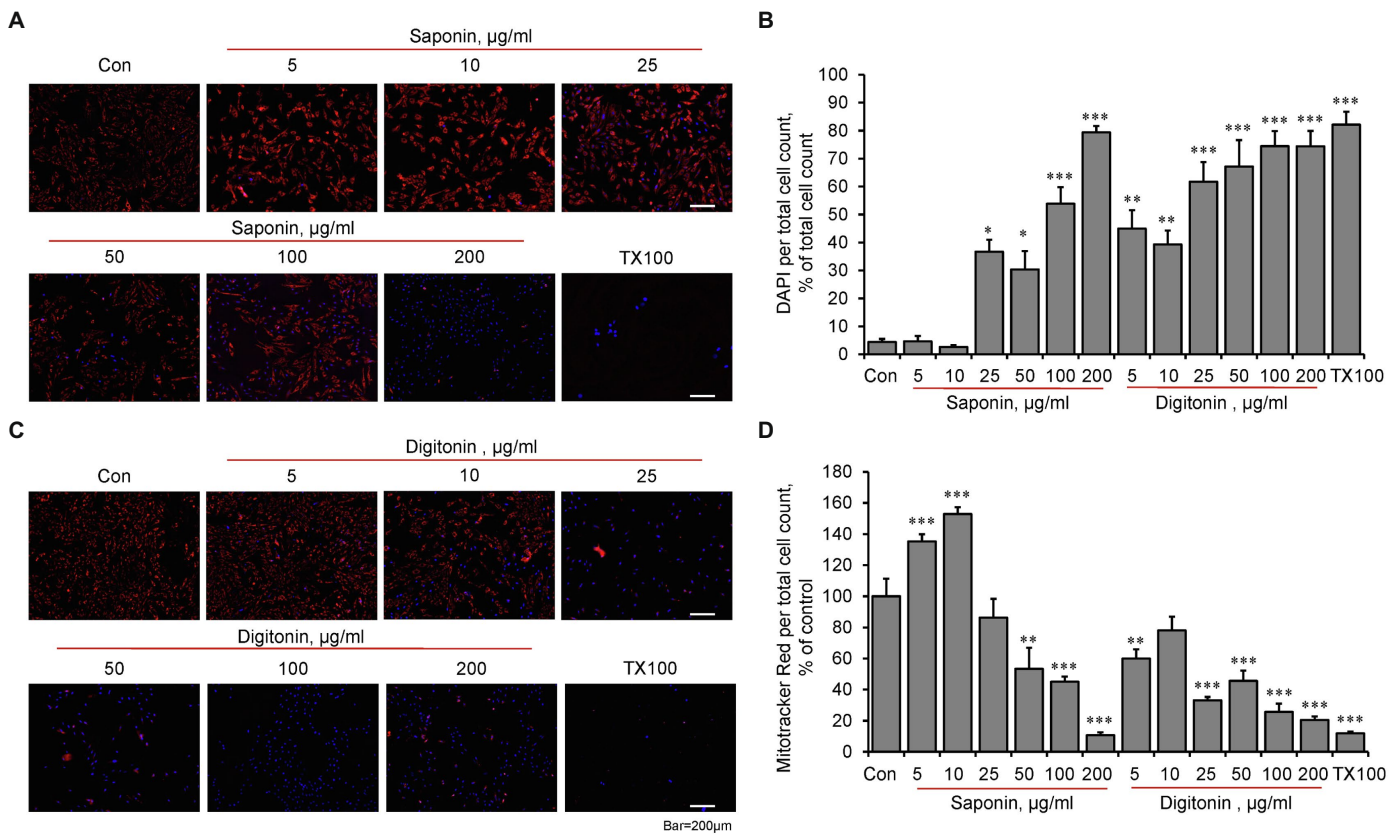
The effects of permeabilization by saponin and digitonin on mitochondria in live H9c2 cells. The cells were grown to 70–80% confluence and incubated with saponin **(A)** or digitonin **(C)** at indicated concentrations for 10 min. Then, the cells were incubated for 30 min with DAPI (*blue*, 300 nM) to visualize the nucleus, as a marker of permeabilization, and with Mitotracker Red (*red*, 30 nM), which is dependent on membrane potential, for visualization of functional mitochondria. Triton X-100 (TX100, 0.1%) was used as a positive control to induce the structural collapse of the cells due to irreversible permeabilization. Images were captured by an Olympus IX73 microscope with LUCPLFLN10X objective using Cellsense Dimension (Olympus) software and quantified using image J for saponin **(B)** and digitonin **(D)**. Data were divided by total cell count and represented as percent of control from live image count. *n* = 3, * *p* < 0.05, ** *p* < 0.01, *** *p* < 0.001 vs. control (Con).

Based on the sensitivity to Ca^2+^ (Figures 1A–C), we used Calcium Green-5N to assess the permeabilization capabilities, potential effects of digitonin and saponin, and the CRC in H9c2 cardioblasts. DAPI, a nuclear staining dye that does not enter non-permeabilized cells, was used to estimate the effectiveness of the detergents to permeabilize the cells. Analysis of permeabilization capabilities demonstrated that both biological detergents at high concentrations have toxic effects on mitochondria, inducing loss of membrane potential (Figures 2A,C,D). The optimal concentrations of the detergents to induce permeabilization of the cells with no effects on mitochondrial functional activity were different for saponin and digitonin. Quantification of DAPI fluorescence to total cells found that saponin levels of 25 μg/ml or higher and all digitonin levels used were capable of permeabilizing the cellular membrane (Figures 2A–C). Analysis of Mitotracker Red displayed mitochondria viability ranging from 5 to 100 μg/ml of saponin as well as digitonin levels between 5 and 10 μg/ml. Saponin effectively permeabilized the cells at concentrations 25, 50, and 100 μg/ml (Figure 2A), whereas optimal permeabilization of the cells by digitonin was observed at 5 and 10 μg/ml (Figure 2C).

**Figure 3:**
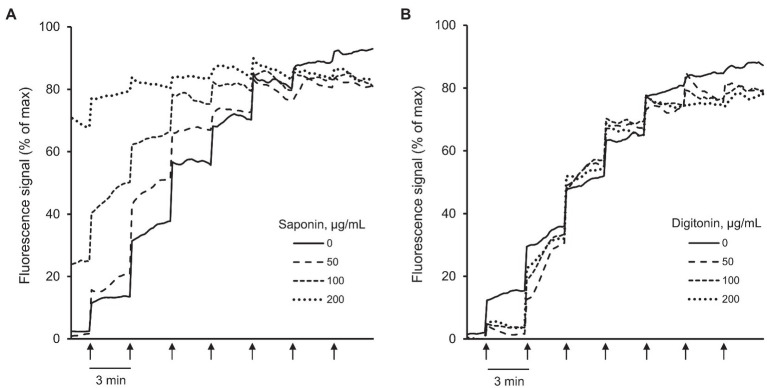
Analysis of interference between Calcium Green-5N and saponin or digitonin. The fluorescence intensity of Calcium Green-5N was measured in the cell-free assay buffer in the presence of 50, 100, and 200 μg/ml saponin **(A)** or digitonin **(B)**. Ca^2+^ was added every 3 min (*arrows*) by increments of 2 nmol/injection. *n* = 6 per group. Saponin at 200 μg/ml had interference with Calcium Green-5N as evidenced by saturated fluorescence signal without added Ca^2+^ **(A)** whereas digitonin at all concentrations had no interference with the dye **(B)**.

To determine whether the detergents have interference with Calcium Green-5N (Figure 3; Supplementary Figures S2A,B), the fluorescence intensity of the dye was measured in the assay buffer (no cells) by adding digitonin or saponin at concentrations of 50, 100, and 200 μg/ml. Saponin at 200 μg/ml demonstrated interference with Calcium Green-5N as evidenced by saturated fluorescence signal in the absence of Ca^2+^ (Figure 3A; Supplementary Figure S2A) whereas digitonin had no interference with the dye at all concentrations (Figure 3B; Supplementary Figure S2B).

### Analysis of the CRC in Permeabilized H9c2 Cardiomyocytes and Isolated Mitochondria

**Figure 4:**
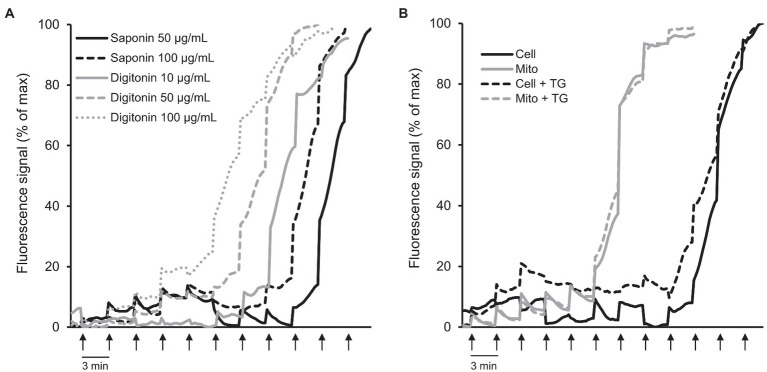
CRC analysis in permeabilized cells *in situ* and isolated mitochondria *in vitro*. **(A)** CRC in H9c2 cells permeabilized by saponin or digitonin. The cells were permeabilized by saponin (50 and 100 μg/ml) or digitonin (10, 50, and 100 μg/ml) for 10 min in sucrose buffer on ice. *n* = 3–6 per group. **(B)** Comparative analysis of CRC in permeabilized cells vs. isolated mitochondria in the presence or absence of 1 μM thapsigargin (TG). Ca^2+^ was added every 3 min (*arrows*) by increments of 1 nmol/injection. 0.6 × 10^6^ H9c2 cells were used for each well. Permeabilized cells were washed off the detergents before the analysis. Curves represent the averages. *n* = 6 per group.

Next, we analyzed the CRC in permeabilized cells *in situ* and in isolated mitochondria *in vitro* to choose the technique which can be used for accurate quantification of mitochondrial CRC in cultured cells. First, we measured the CRC in H9c2 cardiomyocytes permeabilized by saponin or digitonin (Figure 4A; Supplementary Figure S2C). The cells permeabilized by digitonin at all concentrations (10, 50, and 100 μg/ml) had lower CRC than saponin-permeabilized cells. The cells permeabilized with 50 and 100 μg/ml saponin required approximately 9 and 10 nmol Ca^2+^, respectively, to induce a massive release of Ca^2+^ whereas nearly 6, 7, and 8 nmol Ca^2+^ were required to open the PTP/Ca^2+^ release from mitochondria of cells permeabilized by 10, 50, and 100 μg/ml of digitonin, respectively.

Considering the negative effects of digitonin on the CRC of mitochondria (Figure 4A; Supplementary Figure S2C), in the next set of experiments, we used saponin-permeabilized cells for comparison of mitochondrial CRC in the permeabilized cells versus isolated mitochondria. An equal number of H9c2 cells (0.6 × 10^6^) were used for permeabilization of cells and isolation of mitochondria. Notably, mitochondrial mass was not decreased significantly (<7%, *p* < 0.974) in isolated mitochondria in comparison with the permeabilized cells as evidenced by citrate synthase activities in both samples. Results demonstrated that the CRC in permeabilized cells was higher compared to the isolated mitochondria (Figure 4B; Supplementary Figure S2D); 10 nmol Ca^2+^ was required to trigger PTP opening/massive Ca^2+^ release in the cells permeabilized with 50 μg/ml saponin whereas isolated mitochondria started swelling at 6 nmol Ca^2+^ indicating at their lower CRC. Thapsigargin, a non-competitive inhibitor of the SERCA, was used to estimate the contribution of sarcoplasmic reticulum to the CRC of mitochondria. The mitochondrial CRC of saponin-permeabilized cells was slightly reduced in the presence of 1 μM thapsigargin. As expected, it had no effect on the CRC of the isolated mitochondria (Figure 4B; Supplementary Figure S2D).

## Discussion

In this study, we attempted to compare two techniques for the measurement of the CRC of mitochondria in cultured cells. Results showed that mitochondrial CRC measured in permeabilized cells is higher than that in isolated mitochondria. Also, we found the optimal concentrations of the biological detergents saponin and digitonin required for effective permeabilization of the H9c2 cells with no toxic effects on mitochondrial function (ΔΨ_m_).

A few techniques have been developed for the quantification of mitochondrial PTP/CRC *in vivo* in cells and tissues. 2-deoxy[^3^H] glucose (DOG) entrapment technique can be applied to measure PTP opening in isolated perfused hearts *in vivo* (Javadov et al., 2005; Ciminelli et al., 2006). In cultured cells, mitochondrial PTP/CRC can be measured directly in intact cells by calcein, a cell-permeant fluorescent probe, the intensity of which is quenched strongly by metal ions, such as Co^2+^, in the cytosol. Hence, the fluorescence quenching in response to Ca^2+^ release from mitochondria through the PTP can allow quantifying the extent of pore opening (Petronilli et al., 1999; Hernandez et al., 2014; Teixeira et al., 2015). However, the technique for analysis of the PTP by calcein has several weaknesses. First, Co^2+^ is a heavy metal and has toxic effects on cells. Second, the technique does not allow to specify whether calcein quenching occurs due to its release through mitochondrial PTPs or results from Co^2+^ entry into mitochondria. For example, incubation of cells with calcein in combination with a red-fluorescing potentiometric dye demonstrated that cytosolic calcein can be released from normal mitochondria but enters them upon PTP opening (Jones et al., 2002). Third, calcein-AM entering mitochondria is not cleaved in all cell types such as hepatocytes (Lemasters et al., 1998).

Advantages of the technique for quantification of the CRC in permeabilized cells *in situ* compared to isolated mitochondria can be explained with the fact that during permeabilization intracellular structural organization of the cells is preserved, allowing to analyze all subcellular populations of mitochondria (Harisseh et al., 2019). Analysis of several types of primary cells and cell lines revealed that mitochondria within individual cells are morphologically heterogeneous with varying sizes, are differently distributed, and they may even have distinct functions (Collins et al., 2002). In contrast, analysis of PTP opening, mitochondrial swelling, and the CRC in isolated mitochondria have certain limitations. Homogenization and centrifugation during the isolation procedure damage mitochondria and induce their swelling (Morikawa et al., 2014). In addition, isolated mitochondria do not present all populations of mitochondria localized in different subcellular compartments.

A large number of studies used permeabilized cells, particularly cardiomyocytes, to investigate mitochondrial bioenergetics and respiration rates (Eimre et al., 2008; Xu et al., 2014), CRC (Harisseh et al., 2019), membrane potential (Keane et al., 2016), ROS (Pham et al., 2000; Keane et al., 2016), and ions (Na^+^, Ca^2+^; Donoso et al., 1992; Dedkova and Blatter, 2009) *in situ*. Permeabilized cardiomyocytes and cardiac muscle fibers have been shown to maintain natural structural organization and mitochondrial bioenergetics (Kay et al., 1997; Saks et al., 1998), which are consistent with our findings (Supplementary Figure S1). Likewise, intracellular morphology and mitochondrial function of H9c2 cardioblasts remained unaffected by permeabilization (Szalai et al., 2000; Medepalli et al., 2013; Namekata et al., 2017). Mostly, two biological detergents digitonin and saponin were used for the permeabilization of cells. Digitonin disrupts the plasma membrane bilayers by targeting lipid rafts while saponins permeabilize plasma membranes by selectively removing cholesterol from the membranes without affecting membrane proteins. The intracellular environment including the structural and functional integrity of subcellular organelles, and interactions between them remain almost intact in permeabilized cells. Interestingly, thapsigargin had no significant effect on the CRC in permeabilized cells (Figure 4B). This can be explained by the low concentration of ATP (due to its dilution in the assay buffer upon permeabilization), which is not sufficient to activate SERCA and stimulate Ca^2+^ uptake by sarcoplasmic reticulum. Apparently, permeabilization efficacy and toxic effects of saponin and digitonin at given concentrations can be varied for different cell types.

In conclusion, this study highlights the advantages of the quantification of mitochondrial CRC in permeabilized cells compared to isolated mitochondria and establishes optimal concentrations of two biological detergents that are widely used for the permeabilization of cells. The results of the study can be taken into consideration during the quantification of mitochondrial CRC in live cells.

## Data Availability Statement

The original contributions presented in the study are included in the article/Supplementary Material, further inquiries can be directed to the corresponding author.

## Ethics Statement

The animal study was reviewed and approved by the UPR Medical Sciences Campus Institutional Animal Care and Use Committee and conformed by the National Research Council Guide for the Care and Use of Laboratory Animals published by the US National Institutes of Health (2011, eighth edition).

## Author Contributions

SJav and SF conceived the study and wrote the manuscript. SJan and XC-D performed the experiments and contributed the data. All authors contributed to the article and approved the submitted version.

## Funding

This study was supported by the grants from the National Institutes of Health (SC1GM128210 and R25GM061838 to SJav; R01NS104127 and R01AG062572 to SF), the National Science Foundation (2006477 to SJav), and the Pennsylvania Department of Heath Collaborative Research on Alzheimer’s Disease (PA Cure, to SF).

## Conflict of Interest

The authors declare that the research was conducted in the absence of any commercial or financial relationships that could be construed as a potential conflict of interest.

## Publisher’s Note

All claims expressed in this article are solely those of the authors and do not necessarily represent those of their affiliated organizations, or those of the publisher, the editors and the reviewers. Any product that may be evaluated in this article, or claim that may be made by its manufacturer, is not guaranteed or endorsed by the publisher.
