# Supplementary material for: Analysis of Mitochondrial Calcium Retention Capacity in Cultured Cells: Permeabilized Cells Versus Isolated Mitochondria
Source: Front Physiol. 2021 Dec 7;12:773839. doi: 10.3389/fphys.2021.773839 (PMC8688924; doi:10.3389/fphys.2021.773839)
Supplement: Supplementary file 1 [file Data_Sheet_1.PDF]

Supplementary Material

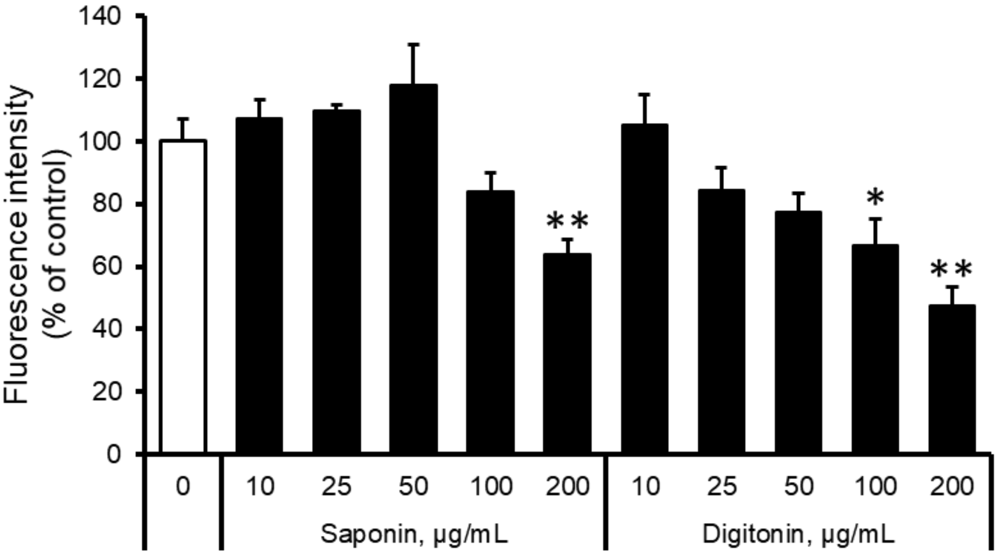

Figure S1. The effects of permeabilization by saponin and digitonin on ATP in live H9c2 cells. The cells were grown to 70-80% confluence and incubated with saponin or digitonin at indicated concentrations for 10 min. Then, the cells were incubated for 15 min with 5 µM Biotracker ATP-Red® (EMD Millipore) to measure ATP levels in live cells. Fluorescence intensity was measured by CLARIOStar microplate reader (BMG Labtech). *p<0.5, **p<0.01, *vs.* control (0). n=4 per group.

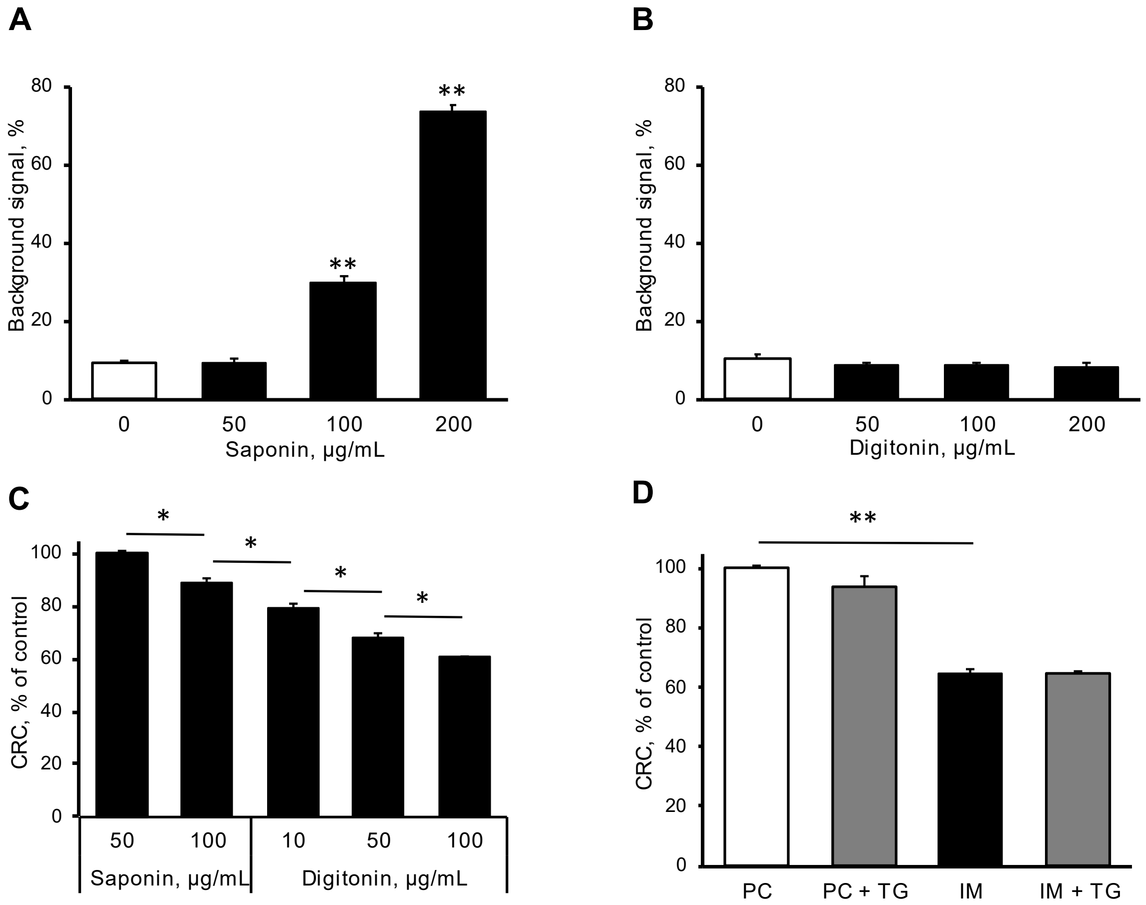

Figure S2. (**A,B**) Analysis of interference between Calcium Green-5N and saponin, or digitonin. The fluorescence intensity of Calcium Green-5N was measured in the cell- and Ca^{2+}- free assay buffer in the presence of 50, 100, and 200 µg/mL saponin (**A**) or digitonin (**B**). **p<0.01 vs. control. n=6 per group. (**C,D**) CRC analysis in permeabilized cells *in situ* and isolated mitochondria *in vitro*. ***C***, CRC in H9c2 cells permeabilized by saponin or digitonin. The cells were permeabilized by saponin (50 and 100 µg/mL) or digitonin (10, 50, and 100 µg/mL) for 10 min in sucrose buffer on ice. n=3-6 per group. ***D***. Comparative analysis of CRC in permeabilized cells (PC) vs. isolated mitochondria (IM) in the presence or absence of 1 µM thapsigargin (TG). 0.6×10^6 H9c2 cells were used for each well. Permeabilized cells were washed off the detergents before the analysis. *p<0.05, **p<0.01. n=6 per group.
